# Effect of apolipoprotein E (APOE) gene polymorphisms on the lipid profile of children being treated for acute lymphoblastic leukemia

**DOI:** 10.1007/s12185-024-03748-6

**Published:** 2024-03-20

**Authors:** Maria Ioannidou, Chrysostomos Avgeros, Elisavet Georgiou, Aliki Papadimitriou-Tsantarliotou, Dimitrios Dimitriadis, Athanasios Tragiannidis, Paraskevi Panagopoulou, Evgenia Papakonstantinou, Assimina Galli-Tsinopoulou, Kali Makedou, Emmanuel Hatzipantelis

**Affiliations:** 1 Pediatric and Adolescent Hematology Oncology Unit, 2nd Department of Pediatrics, Faculty of Health Sciences, School of Medicine, AHEPA University General Hospital, Aristotle University of Thessaloniki, St. Kyriakidi 1, 54621 Thessaloniki, Greece; 2 Laboratory of Biological Chemistry, Faculty of Health Sciences, School of Medicine, Aristotle University of Thessaloniki, Thessaloniki, Greece; 3 Laboratory of Pharmacology, Faculty of Health Sciences, School of Pharmacy, Aristotle University of Thessaloniki, Thessaloniki, Greece; 4 School of Economics, Aristotle University of Thessaloniki, Thessaloniki, Greece; 5 4th Department of Pediatrics, Faculty of Health Sciences, School of Medicine, Papageorgiou General Hospital, Aristotle University of Thessaloniki, Thessaloniki, Greece; 6 Department of Pediatric Oncology, Hippokration Hospital, Thessaloniki, Greece

**Keywords:** Pediatric acute lymphoblastic leukemia, Polymorphisms, Lipoprotein lipase (LpL), Apolipoprotein E (apoE), Lipid profile

## Abstract

**Background:**

Medications used to treat acute lymphoblastic leukemia (ALL), such as l-asparaginase, can cause blood lipid disturbances. These can also be associated with polymorphisms of the lipoprotein lipase (LpL) and apolipoprotein E (APOE) genes.

**Procedure:**

We aimed to investigate the association between lipid profile, certain LpL and APOE gene polymorphisms (rs268, rs328, rs1801177 and rs7412, rs429358 respectively) as well as the risk subgroup in 30 pediatric patients being treated for ALL, compared with 30 pediatric ALL survivors and 30 healthy controls.

**Results:**

The only APOE gene polymorphism with significant allelic and genotypic heterogeneity was rs429358. Further analysis of this polymorphism showed that genotype (CC, CT, or TT) was significantly associated with (1) changes in the lipid profile at the end of consolidation (total cholesterol, LDL, apo-B100, and lipoprotein a) and during re-induction (total cholesterol and apo-B100), and (2) classification in the high risk-ALL subgroup (for CC genotype/C allele presence).

**Conclusions:**

Lipid abnormalities in children being treated for ALL may be associated with the APOE genotype, which is also possibly associated with risk stratification. Further research is needed to confirm the potential prognostic value of these findings.

**Supplementary Information:**

The online version contains supplementary material available at 10.1007/s12185-024-03748-6.

## Introduction

Acute leukemia is the most common malignant disease in childhood and accounts for approximately one-third of all cancer types diagnosed in children aged 0 to 14 years. Its incidence rate in Europe ranges between 42 and 59 cases per million people aged 0–14 years old [1]. Acute lymphoblastic leukemia (ALL) is the most common type of childhood leukemia, representing approximately 75% of all cases [2]. Combination chemotherapy has led to significant improvements in childhood ALL outcome with a 5-year overall survival rate exceeding 90% [3]. One of the key elements of childhood ALL treatment protocols is the use of l-asparaginase (l-ASP) that has increased survival rates since its introduction in the 1960s. l-asparaginase is a substantial and integral component of all protocols developed for children with ALL. Lymphoblastic leukemic cells lack or have low levels of the enzyme asparagine synthetase; therefore, they are unable to produce asparagine on their own and heavily depend on extracellular sources.

Its mechanism of action mainly involves the reduction of the concentration of the amino acid asparagine in both plasma and cerebrospinal fluid (CSF) [4]. Despite the paramount importance of l-ASP in the treatment of leukemia, it is associated with a plethora of serious adverse events such as hypersensitivity reactions, pancreatitis, thrombosis, encephalopathy, and liver dysfunction [5]. In addition, l-ASP has been reported to cause abnormalities in lipid metabolism, ranging from hypocholesterolemia to hypercholesterolemia and hypo- to hypertriglyceridemia during therapy [6].

Single-nucleotide polymorphisms (SNPs) are variations that are widely studied in the context of pharmacogenomics research, as they contribute to the identification of genetic alterations potentially associated with various diseases and conditions. Lipoprotein lipase (LpL) plays a key role in lipid metabolism and mediates the intravascular hydrolysis of triglycerides. Several SNPs located within the coding region of the gene encoding LpL (*LpL* gene) have been associated with diseases and conditions such as atherosclerosis, obesity, dyslipidemia, and Alzheimer’s disease [7]. Apolipoprotein E (ApoE) is one of the structural elements of lipoproteins and plays an important role in the cell uptake of lipids. There are three isoforms of the apoE lipoprotein: ΑpoE2, ΑpoE3, and ΑpoE4, which are encoded by the corresponding combinations of the ε2, ε3, and ε4 alleles of the *APOE* gene, respectively. These alleles (ε2, ε3, and ε4) are the result of certain SNPs described for the *APOE* gene, namely rs429358 and rs741. Genetic variants ε2, ε3, and ε4 and their corresponding protein variants (apoE2, apoE3, and apoE4) have been linked to various risks of dyslipidemia, as well as coronary artery disease [8]. The association between the genetic background of LpL and apoE and the lipid profile of children with ALL treated with l-asparaginase has not been previously studied. The present study aimed to evaluate potential associations between *LpL* and *APOE* genotype or alleles with lipid profile alterations during treatment and also with ALL risk subgroup.

## Methods

The present study was a prospective, case–control study that was conducted in the Pediatric and Adolescent Hematology Oncology Unit of the 2nd Department of Pediatrics of a university-affiliated, tertiary care hospital in Thessaloniki, Greece, between May 2019 and September 2021. It was approved by the Bioethics Committee of the School of Medicine (protocol number: 6.321/29-07-2020). Parents/guardians of all participants were informed about the aims of the study and provided written informed consent, while patients aged  > 12 years provided their written assent.

### Sample collection

Whole blood (in EDTA-containing tubes) and serum samples were collected from all participants, who were divided into three groups; group A included 30 children and adolescents with newly diagnosed ALL, group B included 30 children and adolescents who had completed chemotherapy for ALL more than 6 months ago, and group C included 30 age- and sex-matched healthy children. Patients with ALL participating in the study had no history of autoimmune disease, prior malignancy, or recent infection. The demographic characteristics of the three study groups were comparable.

### Exclusion criteria

Patients with abnormal thyroid function tests at the initial evaluation, those with a family history of hypercholesterolemia, a body mass index (BMI) greater than two standard deviations above average according to the World Health Organization (WHO) reference curves and those that had received corticosteroids or total parenteral nutrition two weeks prior to the evaluation, were excluded from the study.

### Clinical and biochemical parameters

Demographic parameters (age and gender), clinical data (underlying disease and treatment phase), and laboratory test results (total cholesterol, triglycerides, high-density lipoprotein (HDL cholesterol), low-density lipoprotein (LDL cholesterol), apolipoprotein A1, apolipoprotein B100 (apo-B100), lipoprotein a [Lp(a)], glucose, amylase and lipase) were recorded in a predefined case report form (CRF).

### Data collection time points

All patients with ALL underwent chemotherapy according to the ALLIC BFM 2009 treatment protocol [9]. Blood lipid profile was evaluated before the administration of l-ASP on days 0 and 11 and after the administration of l-ASP on days 15, 24, and 33 during induction, Protocol IA for all patients. The stratification into risk groups was based on disease characteristics and response to treatment on Day 33. The lipid profile was reevaluated on Day 60 (at the end of consolidation Protocol ΙΒ). At the end of Protocol IB, patients continued their chemotherapeutic protocol according to risk group (IR or HR), based on their disease characteristics and response to treatment. For IR patients, lipid profile was again assessed on days 8, 16, and 21 of re-induction (Protocol II).

### Genotypic analysis

Genotypic analysis for SNPs of the *LpL* and *APOE* genes was conducted for all 90 study participants. Specifically, genomic DNA was isolated from whole blood using the Monarch Genomic DNA Purification Kit (New England Biolabs, Ipswich, MA, USA). *LpL* rs328 (C > G), rs1801177 (G > A), rs268 (A > G) SNPs, and *APOE* rs7412 (C > T) and rs429358 (T > C) polymorphisms were assessed by RNase H2 enzyme-based amplification (rhAmp) SNP assay (Integrated DNA Technologies, Coralville, USA) using real-time PCR according to the manufacturer’s instructions. Analysis was performed on a StepOnePlus Real Time PCR system (Applied Biosystems). Cycling conditions included a prereading stage of 30 s at 60 °C; enzyme activation for 10 min at 95 °C; 40 cycles of 10 s at 95 °C, 30 s at 60 °C, 20 s at 68 °C; and a final post-reading stage of 30 s at 60 °C.

### Statistical analysis

The sample size for the present study was determined based on a previous pilot study in which an effect size of 0.5 was added to a power (1 − *b*) = 0.95 and a level of significance equal to *α* = 0.05. Data were analyzed using Student’s *t*-test for independent samples (G*power 3.1 for Windows software). The optimal sample size was calculated no less than 90 participants (i.e., 30 for each group). The chi-square test of independence was performed to detect a possible relationship between genotypes and alleles with risk subgroup. Independent samples *t*-test was applied to examine differences in the studied lipid parameters among different alleles/genotypes subgroups. The comparison of the genotypic and allelic patterns of the polymorphisms of interest in the study was performed using the dominant, the recessive, and the allelic models. In the dominant model, a homozygous wild-type genotype was used as a reference to investigate the prevalence of genotypes associated with mutant alleles. In the recessive model, genotypes associated with wild-type alleles were used as a reference to investigate the prevalence of the mutant homozygous genotype. Finally, in the allelic model, the wild-type allele was used as a reference to investigate the prevalence of the mutant allele. IBM SPSS v. 28 software package was used, and all tests are provided at a 5% level of significance.

## Results

### Demographic characteristics

A total of 30 patients were recruited in Group A (19 female; age range: 12 months–16 years, mean (± SD): 6.55 (± 4.63) years). Twenty-four patients were diagnosed with B-ALL and six with T-ALL. Among them, 23 patients were eventually classified as IR and 7 as HR. In Group B, 30 off-treatment patients (survivors) (19 female; age range: 3–16 years, mean age (± SD): 7.06 (± 3.64) years) were recruited. In Group C, 30 healthy children (19 female; age range: 1–16 years, mean (± SD): 7.3 (± 4.06) years) were included. The main demographic characteristics of each group are shown in Table 1.

**Table 1:** Demographic and disease characteristics of study participants (groups A, B, and C)

	Group A	Group B	Group C	*p*-value
Type of subject	New ALL diagnosis	Control group 1 (survivors)	Control group 2 (healthy)	
Number	30	30	30	
Gender	11:19	11:19	11:19	ns
M:F				
*N*% M	37%	37%	37%	
*N*% F	63%	63%	63%	
Age (years) [mean(± SD)]	6.55 (± 4.63)	7.06 (± 3.64)	7.3 (± 4.06)	ns
Risk group	23 ΙR 7 HR	na	na	na

*M:* male, *F:* female, *IR:* intermediate risk, *HR:* high risk, *ns:* nonsignificance, *na*: not applicable

### Genetic analyses

Allelic and genotypic patterns of polymorphisms rs429358, rs7412 (of *APOE* gene), and rs328, rs1801177, and rs268 (of *LpL* gene) were studied.

For *LpL*, no SNP had significant heterogeneity for the study cohort. For APOE, all patients in group A had CC genotype for rs7412 and only for rs429358 showed significant heterogeneity and was further examined. Results according to risk group are shown in Table 2. Thus, classification into carriers of apoE ε2, ε3, and ε4 alleles and the corresponding isoforms was solely influenced by the genotypic profile of rs429358 (ε3/ε3: rs429358 TT + rs 7412 CC, ε3/ε4: rs429358 TC + rs7412 CC, and ε4/ε4: rs429358 CC + rs7412 CC). Statistical analysis according to the dominant model showed that the frequencies of TT vs. TC + CC genotypes did not have a significant difference between HR and IR patients OR = 0.18, 95% CI 0.01–1.75, *p* = *0.113*). We, therefore, conclude that the relative probability for an individual with the ε3/ε3 isoform to be included in the HR group is not statistically different from the relative probability for a patient with the ε3/ε3 isoform to be classified as IR (Table 2).

**Table 2:** Analysis of allelic and genotypic profile of rs429358 for Group A patients

	HR patients (*n* = 7)	ΙR patients (*n* = 23)	Isoforms	*p*-value	Unadjusted OR (95% CI)
Genotype *n* (%)					
TT	1 (14%)	11 (48%)	12 ε3/ε3		
TC	1 (14%)	8 (35%)	9 ε3/ε4		
CC	5 (72%)	4 (17%)	9 ε4/ε4		
Dominant model					
TT	1 (14%)	11 (48%)		*p* = *0.113*	1 (reference) 0.18 (0.01–1.75)
TC + CC	6 (86%)	12 (52%)			
Recessive model					
TC + TT	2 (29%)	19 (83%)		*p* = *0.006*	1(reference) 11.875 (1.66–84.51)
CC	5 (71%)	4 (17%)			
Allelic model					
T	3/14 (21%)	30/46 (65%)		*p* = *0.004*	1 (reference) 7.14 (1.69–33.33)
C	11/14 (79%)	16/46 (35%)			

*HR:* high risk, *IR:* intermediate risk

Moreover, in the recessive model (TC + TT vs. CC) and the allelic model, both the CC genotype or the C allele, respectively, showed a higher distribution in HR patients compared to IR and their presence increased the probability of patients being classified into HR group [(OR = 11.875; 95% CI: 1.66–84.51, *p* = *0.006*) and (OR = 7.14; 95% CI 1.69–33.33, *p* = *0.004*), respectively]. The same observation applies to individuals with the ε4/ε4 isoform (Table 2).

### Association of genotypic profile of the rs429358 polymorphism with the lipid profile of children with ALL during their treatment

The study also investigated the association of the genotypic profile of rs429358 with all measured variables at the scheduled time points during treatment. For each time point, two comparisons were performed based on the dominant and the recessive genotypic models. There was no statistical difference for any of the measured variables on days 0, 11, 15, 24, and 33 (Protocol IA). During induction, no patient had to interrupt their treatment because of lipid abnormalities, whereas regarding seven HR patients, three had the t(9;22) translocation (BCR-ABL) and the remaining four had a poor BM response (BM M2/M3) on Day 33. However, a statistically significant difference was noted on Day 60 for the following variables: total cholesterol, LDL, apoB-100, and lipoprotein a (Table 3). Specifically, children with the TT genotype presented significantly higher total cholesterol and LDL cholesterol και apoB-100 levels compared to children with the TC + CC genotypes (*p* = *0.033*, *p* = *0.009,* and *p* = *0.012,* respectively). The same applies to ε3/ε3 isoforms.

**Table 3:** Comparison of the lipid profile (total cholesterol, LDL, apoB-100, and lipoprotein a) according to rs429358 genotype at Day 60 (t-test/two independent samples) [Group A]

Total cholesterol (mg/dL)		
	Mean (± SD)	*p*-value
*Dominant model*		
TT (*N* = 12)	164 (± 34)	*p* = 0.033
TC + CC (*N* = 18)	134 (± 34)	
*Recessive model*		
TC + TT (*N* = 21)	153 (± 34)	*p* = 0.191
CC (*N* = 9)	133 (± 39)	
LDL (mg/dL)		
	Mean (± SD)	*p*-value
*Dominant model*		
TT (*N* = 12)	102 (± 25)	*p* = 0.009
TC + CC (*N* = 18)	73 (± 27)	
*Recessive model*		
TC + TT (*N* = 21)	88 (± 32)	*p* = 0.370
CC (*N* = 9)	77 (± 22)	
ApoB-100 (mg/dL)		
	Mean (± SD)	*p*-value
*Dominant model*		
TT (*N* = 12)	94 (± 28)	*p* = 0.012
TC + CC (*N* = 18)	71 (± 18)	
*Recessive model*		
TC + TT (*N* = 21)	86 (± 27)	*p* = 0.077
CC (*N* = 9)	68 (± 15)	
Lipoprotein A (nmol/l)		
	Mean (± SD)	*p*-value
*Dominant model*		
TT (*N* = 12)	41 (± 63)	*p* = 0.124
TC + CC (*N* = 18)	10 (± 16)	
*Recessive model*		
TC + TT (*N* = 21)	(30 (± 50)	*p* = 0.028
CC (*N* = 9)	9 (± 4)	

*LDL:* low-density lipoprotein cholesterol, *ApoB-100:* apolipoprotein B100

Concerning lipoprotein a, children with CC genotype had significantly lower values than children with TC + TT genotypes (*p* = *0.028*). The same observation also applies to individuals with ε4/ε4 isoforms. The results for cholesterol, LDL, apoB-100, and lipoprotein a on Day 60 are also presented in Table 3.

Analysis was also performed for the 23 IR patients during Protocol II at scheduled time points (Table 4). The study was not feasible for HR patients due to the small sample size. A statistically significant difference in levels of total cholesterol was observed on Day 8 and Day 16. IR children with the TT genotype presented significantly higher cholesterol levels than children with TC + CC genotypes (*p* = *0.027*, *p* = *0.016*, respectively). On Day 21, there was a statistically significant difference for apoB-100 levels. Specifically, IR patients with TT genotype had significantly higher apoB-100 concentrations than those with TC + CC (*p* = *0.013*). Likewise, we can conclude the same regarding the patients with ε3/ε3 isoforms.

**Table 4:** Comparison of the lipid profile (levels of total cholesterol and apo-B100), according to the rs429358 polymorphism genotype pattern during Protocol II [Group A]

Total cholesterol Day 8 (mg/dL)		
	Mean (± SD)	*p*-value
*Dominant model*		
TT (*N* = 12)	186 (± 40)	*p* = 0.027
TC + CC (*N* = 18)	155 (± 27)	
*Recessive model*		
TC + TT (*N* = 21)	173 (± 39)	*p* = 0.366
CC (*N* = 9)	155 (± 8)	
Total cholesterol Day 16 (mg/dL)		
	Mean (± SD)	*p*-value
*Dominant model*		
TT (*N* = 12)	206 (± 67)	*p* = 0.016
TC + CC (*N* = 18)	147 (± 33)	
*Recessive model*		
TC + TT (*N* = 21)	182 (± 62)	*p* = 0.456
CC (*N* = 9)	143 (± 31)	
Apo-B100 Day 21 (mg/dL)		
	Μean (± SD)	p-value
*Dominant model*		
TT (*N* = 12)	122 (± 44)	*p* = 0.013
TC + CC (*N* = 18)	86 (± 6)	
*Recessive model*		
TC + TT (*N* = 21)	106 (± 40)	*p* = 0.785
CC (*N* = 9)	86 (± 2)	

*ApoB-100:* apolipoprotein B100

### Association of rs429358 with the lipid profile of the control groups (survivors and healthy children)

Finally, we studied the genotypic profile of the rs429358 in relation to the measured variables in the two control groups. Two comparisons were performed using the dominant and recessive models for both control groups at Day 0. No statistically significant difference was observed.

## Discussion

The present study showed that the lipid profile of children with ALL was not affected by any of the studied polymorphisms except rs429358 of the *APOE* gene. More specifically, no statistically significant difference was observed for any of the measured variables in all study groups on Day 0. Furthermore, in Group A, even during the administration of l-asparaginase during Protocol IA, no change was shown. In contrast, we documented an alteration in the lipid profile on Day 60 of Protocol IB, as well as on days 8, 16, and 21 of Protocol II for Group A, IR patients. In addition, children carrying the ε3 allele of the *APOE* gene had significantly higher cholesterol and LDL και apoB-100 levels compared to children carrying one or two ε4 alleles on Day 60. Regarding lipoprotein α, children with ε4/ε4 genotype had significantly lower levels than children with ε3/ε4. During Protocol II (re-induction) on days 8 and 16, children with ε3/ε3 genotype presented significantly higher cholesterol levels than ε3/ε4, ε4/ε4; on Day 21, ε3/ε3 patients were found to have significantly higher apoB-100 values. Finally, the CC genotype and the C allele were found more frequently in HR patients when compared with IR patients.

Polymorphisms in the *LpL* and *APOE* genes have been linked with altered lipid profiles in various populations. ALL is the most common malignancy in children and is also known to be associated with abnormal lipid profile. l-asparaginase is a chemotherapeutic agent invariably used in the treatment of ALL and known to cause alterations in lipid metabolism. However, the association of the genetic background of LpL and apoE with the lipid profile in children with ALL receiving asparaginase has not been previously studied.

The study’s findings regarding the ε4 allele and its association with total cholesterol and LDL levels appear to differ from previous reports. Previous studies have revealed that genotypes containing the ε4 allele are associated with increased levels of total cholesterol and LDL [10]. The presence of apoΕ4 is associated with higher levels of apoB and total and LDL cholesterol [11] due to its preference for VLDL and higher VLDL lipolytic activity compared to apoE3 [12]. In a meta-analysis of 48 studies, it was shown that the ɛ4 allele was a significant risk factor for coronary heart disease [13]. Another meta-analysis of 59 studies including 13,843 subjects revealed that the ε4 and ε2 alleles of the *apoE* gene are risk factors for hyperlipidemia [14].

However, there are limited studies related to the effect of *apoE* polymorphisms on the lipid profile in children. In 81 obese children, the ratios of total cholesterol/HDL and LDL/HDL were found to be higher in the E4/E3 genotype compared to those with E3/Ε3 and E2/Ε3 [15]. In a sample of 647 African American and 573 White Caucasian girls aged 9–10 years, the ε2 allele was significantly associated with lower mean LDL and apoB levels, whereas the ε4 allele was associated with higher LDL and apoB levels in both racial groups [16]. However, some studies report that HDL concentrations were significantly lower in carriers of the ε4 allele and higher in carriers of the ε2 allele [17, 18]. Although there are numerous studies examining the lipid profile of children treated for ALL [19, 20], this research about the association between *LpL* and *APOE* gene polymorphisms and lipid levels in these patients is unique.

*LpL* gene polymorphism rs328 has been associated with a reduced risk for cardiovascular disease [21]. In a meta-analysis, the authors evaluated the association between rs268 and rs1801177 of the *LpL* gene and the risk for coronary heart disease. Individuals with both polymorphisms had lower levels of HDL cholesterol and higher levels of triglycerides compared to individuals without the polymorphisms [22]. In our cohort, there was no significant heterogeneity for any of the studied *LpL* gene SNPs, so no further testing was possible with regard to the lipid profile and subsequent risk.

The fact that the lipid profile of ALL patients gradually changes after treatment with l-ASP is well known and has previously reported [6, 23]. However, not all patients exhibit such changes, and the examination of the abovementioned polymorphisms and their association with the complex lipid profile changes during ALL treatment provides a valuable insight into the pathogenesis of these alterations.

As mentioned above, the only SNP that showed significant heterogeneity in our study in terms of its genotypic and allelic pattern was rs429358 and we also found a possible association with the risk stratification. More specifically, the CC genotype and the C allele were found more frequently in HR patients when compared to IR patients, indicating an increased probability of having a higher risk of leukemia. This finding has not been previously studied or documented in the literature. Further research is needed to confirm this association and investigate the underlying mechanisms. If confirmed, it could be a helpful tool for risk stratification of children with ALL. It is noteworthy that multiple factors, including genetic alterations and response to treatment, are taken into account when assigning risk group in pediatric ALL. A possible explanation could be that the presence of certain genotypes and the related lipid alterations contribute to resistance to chemotherapy or increased risk for adverse events that delay treatment.

Although the study sample size was computed as sufficient, ALL has many subtypes (according to immunophenotype, genetics, and risk group) that prevented further subanalyses and adjustments and this could be considered as a study limitation. Also, our data were not adjusted for other factors possibly affecting the lipid profile such as dietary habits. In contrast, a strength of our study is that we investigated a parameter that has not been previously examined and found some interesting and promising results.

In conclusion, it seems that lipid abnormalities during treatment in children with ALL could be associated with the *ApoE* genotype which is also possibly associated with risk stratification. The evaluation of the described polymorphisms was conducted for the first time in children of Greek origin, especially for the first time in children with leukemia worldwide. However, larger studies with more patients from varied ethnic backgrounds are needed to confirm our results.

### Supplementary Information

Below is the link to the electronic supplementary material.

Supplementary file1 (DOCX 21 KB)

## Abbreviations

ALL: Acute lymphoblastic leukemia
Apo-B100: Apolipoprotein B100
ApoE: Apolipoprotein E
BMI: Body mass index
CRF: Case report form
CSF: Cerebrospinal fluid
HDL cholesterol: High-density lipoprotein cholesterol
HR: High risk
IR: Intermediate risk
l-ASP: L-asparaginase
LDL cholesterol: Low-density lipoprotein cholesterol
Lp(a): Lipoprotein a
LpL: Lipoprotein lipase
SNPs: Single-nucleotide polymorphisms
VLDL: Very-low-density lipoprotein
WHO: World Health Organization
