# Supplementary material for: Effect of apolipoprotein E (APOE) gene polymorphisms on the lipid profile of children being treated for acute lymphoblastic leukemia
Source: Int J Hematol. 2024 Mar 20;119(6):755–61. doi: 10.1007/s12185-024-03748-6 (PMC11140815; doi:10.1007/s12185-024-03748-6)
Supplement: Supplementary file 1 — Supplementary file1 (DOCX 21 KB) [file 12185_2024_3748_MOESM1_ESM.docx]

**Supplementary Material**

1. **Frequencies of genotyping variants of rs 7412 (APOE) and rs 268, rs 328 and rs 1801177 (LpL) for Group A patients**

| ***rs 7412 (C>T)*** |  |
| --- | --- |
| **Genotype** | **Patients (n=30)** |
| CC | 30 (100%) |
| TC | 0 (0%) |
| TT | 0 (100%) |

| **Genotype** | **rs 268 (A>G)** | **rs 328 (C>G)** | **rs 1801177 (G>A)** |
| --- | --- | --- | --- |
| **CC** |  | 26 (87%) | 0 (0%) |
| **CG** |  | 4 (13%) | 0 (0%) |
| **GG** | 0 (0%) | 0 (0%) | 30 (0%) |
| **AA** | 30 (100%) |  |  |
| **GA** | 0 (0%) |  |  |

1. **Lipid differentiation according to genotype in all 3 groups at baseline (Day 0)**

| **Value** | **CC vs TT + TC**  **p value**  **(CC: mean**$\boldsymbol{\pm}$**std vs TT+TC: mean**$\boldsymbol{\pm}$**std)** | **TT vs CC + TC**  **p value**  **(TT: mean**$\boldsymbol{\pm}$**std vs CC+TC: mean**$\boldsymbol{\pm}$**std)** |
| --- | --- | --- |
| Total cholesterol (mg/dl) | Group A: 0.205  (151.78$\pm$36.32 vs 134.48$\pm$32.29)  Group B: 0.430  (150.67$\pm$16.29 vs 161.48$\pm$22.59)  Group C: 0.648  (127$\pm$ - vs 143.07$\pm$34.22) | Group A: 0.166  (129.08$\pm$31.38 vs 146.72$\pm$34.49)  Group B:0.956  (160.19$\pm$26.35 vs 160.64$\pm$16.86)  Group C: 0.468  (137.64$\pm$27.51 vs 146.81$\pm$38.78) |
| Triglycerides (μg/dl) | Group A: 0.179  (205.67$\pm$69.83 vs 165.52$\pm$74.31)  Group B: 0.426  (57.50$\pm$12.28 vs 68.76$\pm$23.54)  Group C: 0.071  (137.00$\pm$ - vs 70.83$\pm$34.73) | Group A: 0.611  (168.92$\pm$81.75 vs 183.33$\pm$70.55)  Group B: 0.697  (69.19$\pm$28.51 vs 65.86$\pm$14.59)  Group C: 0.206  (82.07$\pm$45.34 vs 65.13$\pm$24.67) |
| HDL (mg/dl) | Group A: 0.305  (21.56$\pm$5.57 vs 25.76$\pm$11.42)  Group B: 0.247  (50.50$\pm$7.76 vs 58.57$\pm$11.38)  Group C: 0.324  (61.00$\pm$ - vs 47.41$\pm$13.29) | Group A: 0.780  (25.25$\pm$14.19 vs 24.00$\pm$6.60)  Group B: 0.561  (58.88$\pm$12.70 vs 56.43$\pm$9.60)  Group C: 0.181  (44.36$\pm$15.88 vs 50.94$\pm$10.08) |
| LDL (mg/dl) | Group A: 0.365  (88.67$\pm$30.54 vs 77.43$\pm$30.67)  Group B: 0.738  (88.00$\pm$8.00 vs 82.85$\pm$25.93)  Group C: 0.427  (100.00$\pm$ - vs 78.48$\pm$26.27) | Group A: 0.288  (73.42$\pm$33.64 vs 85.72$\pm$28.22)  Group B: 0.516  (80.56$\pm$30.64 vs 86.57$\pm$16.02)  Group C: 0.427  (75.07$\pm$17.44 vs 82.81$\pm$32.01) |
| Apo-AI (mg/dl) | Group A: 0.355  (74.61$\pm$18.28 vs 83.41$\pm$25.26)  Group B: 0.274  (138.50$\pm$4.44 vs 153.35$\pm$22.66)  Group C: 0.723  (120.00$\pm$ - vs 129.34$\pm$25.67) | Group A: 0.555  (77.25$\pm$30.33 vs 83.11$\pm$18.07)  Group B: 0.671  (153.50$\pm$23.84 vs 150.00$\pm$20.33)  Group C: 0.282  (123.64$\pm$28.11 vs 133.75$\pm$22.36) |
| apoB100 (mg/dl) | Group A: 0.365  (87.11$\pm$15.00 vs 95.67$\pm$25.82)  Group B: 0.792  (84.00$\pm$1.73 vs 81.37$\pm$19.87)  Group C: 0.975  (80.00$\pm$ - vs 80.76$\pm$24.04) | Group A: 0.223  (99.50$\pm$29.99 vs 88.83$\pm$16.94)  Group B: 0.181  (78.06$\pm$19.70 vs 85.71$\pm$9.47)  Group C: 0.959  (80.50$\pm$13.83 vs 80.94$\pm$30.22) |
| Lipoprotein a [Lp(a)] (nmol/l) | Group A: 0.322  (24.89$\pm$17.19 vs 36.71$\pm$46.88)  Group B: 0.412  (3.75$\pm$3.32 vs 24.10$\pm$41.64)  Group C: 0.719  (9.00$\pm$ - vs  25.98$\pm$45.86) | Group A: 0.440  (40.27$\pm$51.57 vs 28.42$\pm$31.57)  Group B: 0.718  (24.59$\pm$38.28 vs 19.18$\pm$42.99)  Group C: 0.491  (19.21$\pm$31.25 vs 30.84$\pm$55.06) |
| Amylase (U/l) | Group A: 0.260  (35.83$\pm$9.32 vs 59.45$\pm$60.74)  Group B: 0.646  (51.17$\pm$13.33 vs 58.17$\pm$25.48)  Group C: 0.266  (91.00$\pm$ - vs 62.10$\pm$25.01) | Group A: 0.304  (40.25$\pm$18.46 vs 60.44$\pm$64.77)  Group B: 0.799  (58.56$\pm$28.39 vs 56.22$\pm$20.06)  Group C: 0.125  (55.50$\pm$25.47 vs 69.69$\pm$23.64) |
| Lipase (U/l) | Group A: 0.903  (92.67$\pm$202.49 vs 81.76$\pm$228.93)  Group B: 0.121  (25.00$\pm$6.08 vs 20.41$\pm$4.60)  Group C: 0.915  (20.00$\pm$ - vs  19.48$\pm$4.73) | Group A: 0.351  (38.75$\pm$71.96 vs 115.89$\pm$274.09)  Group B: 0.380  (20.13$\pm$5.37 vs 21.71$\pm$4.21)  Group C: 0.440  (18.79$\pm$5.06 vs 20.13$\pm$4.32) |
| Glucose (mg/dl) | Group A: 0.206  (82.56$\pm$9.21 vs 94.86$\pm$27.60)  Group B: 0.177  (102.00$\pm$11.53 vs 88.67$\pm$8.90)  Group C: 0.366  (78.00$\pm$ - vs  85.55$\pm$8.08) | Group A: 0.662  (88.75$\pm$29.97 vs 92.78$\pm$20.10)  Group B: 0.489  (88.81$\pm$11.19 vs 91.36$\pm$8.23)  Group C: 0.652  (84.57$\pm$8.48 vs 85.94$\pm$7.90) |

*The standard deviation for subgroup with genotype CC in group C cannot be calculated because the sample size is just n=1.*
